# Targeting and Efficacy of Novel mAb806-Antibody-Drug Conjugates in Malignant Mesothelioma

**DOI:** 10.3390/ph13100289

**Published:** 2020-10-02

**Authors:** Puey-Ling Chia, Sagun Parakh, Ming-Sound Tsao, Nhu-An Pham, Hui K. Gan, Diana Cao, Ingrid J. G. Burvenich, Angela Rigopoulos, Edward B. Reilly, Thomas John, Andrew M. Scott

**Affiliations:** 1 Tumour Targeting Laboratory, Olivia Newton-John Cancer Research Institute, Melbourne, Victoria 3084, Australia; chiapueyling@yahoo.com.sg (P.-L.C.); sagun.parakh@onjcri.org.au (S.P.); hui.gan@onjcri.org.au (H.K.G.); diana.cao@onjcri.org.au (D.C.); ingrid.burvenich@onjcri.org.au (I.J.G.B.); angela.rigopoulos@onjcri.org.au (A.R.); 2 Faculty of Medicine, University of Melbourne, Melbourne, Victoria 3010, Australia; 3 Department of Medical Oncology, Austin Health, Melbourne, Victoria 3084, Australia; 4 School of Cancer Medicine, La Trobe University, Plenty Rd &, Kingsbury Dr, Bundoora, Victoria 3086, Australia; 5 Princess Margaret Cancer Centre, University Health Network, Toronto, ON M5G 2C1, Canada; ming.tsao@uhn.ca (M.-S.T.); Nhu.pham@uhn.ca (N.-A.P.); 6 AbbVie Inc., North Chicago, IL 60064, USA; ed.reilly@abbvie.com; 7 Department of Molecular Imaging and Therapy, Austin Health, Melbourne, Victoria 3084, Australia

**Keywords:** EGFR, malignant mesothelioma, 806-ADC, ^89^Zr-ch806

## Abstract

Epidermal growth factor receptor (EGFR) is highly overexpressed in malignant mesothelioma (MM). MAb806 is a novel anti-EGFR antibody that selectively targets a tumor-selective epitope. MAb806-derived antibody drug conjugates (ADCs), ABT-414, ABBV-221 and ABBV-322, may represent a novel therapeutic strategy in MM. EGFR and mAb806 epitope expressions in mesothelioma cell lines were evaluated using an array of binding assays, and the in vitro cell effects of ABT-414 and ABBV-322 were determined. In vivo therapy studies were conducted in mesothelioma xenograft and patient-derived xenograft (PDX) tumor models. We also performed biodistribution and imaging studies to allow the quantitative targeting of MM by mAb806 using a ^89^Zr-labeled immunoconjugate—ch806. A high EGFR expression was present in all mesothelioma cell lines evaluated and mAb806 binding present in all cell lines, except NCIH-2452. ABT-414 and ABBV-322 resulted in significant tumor growth inhibition in MM models with high EGFR and mAb806 epitope expressions. In contrast, in an EGFR-expressing PDX model that was negative for the mAb806 epitope, no growth inhibition was observed. We demonstrated the specific targeting of the mAb806 epitope expressing MM tumors using ^89^Zr-based PET imaging. Our data suggest that targeting EGFR in MM using specific ADCs is a valid therapeutic strategy and supports further investigation of the mAb806 epitope expression as a predictive biomarker.

## 1. Introduction

Malignant mesothelioma (MM) is a fatal asbestos-associated malignancy that is associated with significant morbidity and mortality. The five-year survival rate of patients diagnosed with MM is less than 5%, and standard frontline treatments have largely been unsuccessful in significantly improving the outcomes. In addition, there are no validated treatments beyond first-line therapy. With the incidences expected to rise [1,2], new therapeutic strategies are urgently needed.

MM is typically characterized by the inactivation of tumor-suppressor genes; however, unlike in non-small cell lung cancer, activating oncogenic mutations are rare. The epidermal growth factor receptor (EGFR), a member of the ErB family, is frequently overexpressed in the majority of MM patients [3,4,5]. However, to date, the therapeutic targeting of EGFR in MM using small molecule inhibitors and monoclonal antibodies have failed to achieve clinical responses or have raised safety concerns [6,7,8,9,10,11]. The lack of *EGFR* kinase domain mutations and *EGFR* gene amplification in MM may explain this lack of response. These results suggest that targeting EGFR by simply abrogating receptor binding or the kinase domain, in the absence of kinase mutations, is ineffective. 

The monoclonal antibody 806 (mAb806) is a novel anti-EGFR antibody that selectively targets a unique epitope of the EGFR only exposed on overexpressed, mutant or ligand-activated forms of the EGFR [12,13]. ABT-806, the humanized form of mAb806, has minimal normal tissue binding and is shown to be well-tolerated [14], representing an attractive therapeutic strategy for use as an antibody-drug conjugate (ADC) in tumors that overexpress EGFR. ADCs generated by conjugating ABT-806 to a cytotoxic payload include ABT-414 (Depatuxizumab Mafodotin; Depatux-M), which has the interchain cysteines of mAb806 conjugated to a potent microtubule inhibitor, monomethyl auristatin F (MMAF), through a noncleavable maleimidocaproyl (mc) linker (mc-MMAF (mafodotin)) with an average drug–antibody ratio (DAR) of 3.8 [15]. Depatux-M has shown efficacy in patients with *EGFR*-amplified recurrent glioblastoma [16,17]. ABBV-221 is comprised of an affinity-matured ABT-806 conjugated to MMAE by a valine-citrulline linker and has a higher affinity for overexpressed EGFR than ABT-414 [18]. ABBV-221 was evaluated in a phase I clinical trial in patient EGFR-expressing solid tumors [19,20]. ABBV-322 is another mAb806-ADC derivative, comprised of the nonaffinity matured version of ABT-806 engineered to include an S238C point mutation that permits site-specific conjugation to a DNA minor groove crosslinking agent, pyrrolobenzodiazepine (PBD), via a cathepsin-cleavable Val-Ala linker with a DAR of two [15,18,21,22] (Supplementary Figure S1).

In this study, we evaluated the antitumor effects of these mAb806-derived ADCs in vivo using MM cell line xenografts and patient-derived xenografts (PDX). We also performed quantitative biodistribution and imaging of radiolabeled mAb806 to establish tumor drug concentrations in vivo and imaging properties of ^89^Zr-ch806, a chimeric form of mAb806 (ch806). Ch806 demonstrated a high affinity for the mAb806 epitope identical to mAb806, as well as similar tumor growth inhibition in vivo as mAb806 [23].

## 2. Results

### 2.1. EGFR and mAb806 Epitope Expression

**Figure 1:**
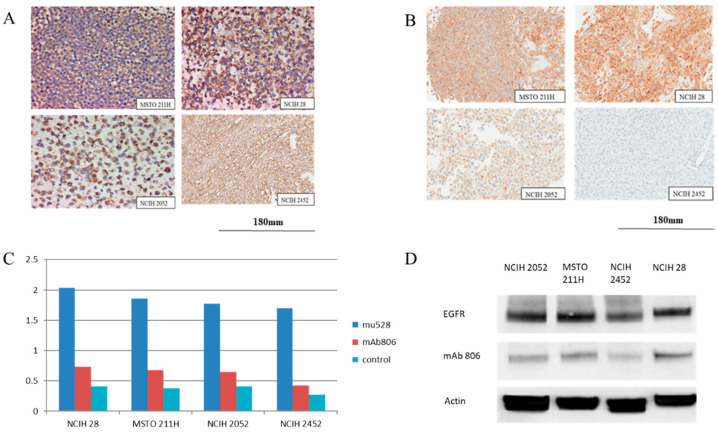
(**A**) Presence of epidermal growth factor receptor (EGFR) immunoreactivity in all 4 mesothelioma cell lines (MSTO-211H, NCIH-28, NCIH-2052 and NCIH-2452). (**B**) Three out of 4 malignant mesothelioma (MM) cell lines (NCIH-28, NCIH-2052 and MSTO-211H) demonstrated mAb806 epitope immunoreactivity. Expression of the mAb806 epitope on immunohistochemical staining (IHC) was considered to be positive if greater or equal to 5% of the cells were stained. (**C**) Comparative bar graphs demonstrating the mean log shift (from triplicates) in fluorescence from the control for each of the 4 mesothelioma cell lines illustrating the relative expression of EGFR (mu528) (dark blue), mAb806 (red) and immunoglobulin G(IgG) idiotype control (light blue). (**D**) Western blot analysis of EGFR and mAb806.

EGFR and mAb806 epitope expression were determined in MSTO-211H, NCIH-28, NCIH-2052 and NCIH-2452 mesothelioma cell lines in a series of binding assays by immunohistochemistry, Fluorescence-activated cell sorting (FACS) and Western blot analysis (Figure 1). Strong EGFR immunoreactivity, when measured by immunohistochemical (IHC) staining using the pan-EGFR antibody mAb528, was present in all four mesothelioma cell lines, as previously reported [24], with median H scores of 150, 210, 125 and 230, respectively. Consistent with the IHC results, strong binding on the FACS and a high EGFR expression was seen on the Western blot.

As expected, mAb806 staining levels were less than that of mAb528 in these cell lines but was clearly seen in all cell lines except NCIH-2452, which showed a lack of mAb806 epitope staining. The median H scores for mAb806 staining were 120, 175, 80 and <5 for MSTO-211H, NCIH-28, NCIH-2052 and NCIH-2452, respectively. Negligible binding was seen on the FACS, and a weak mAb806 expression was seen on the Western blot analysis (Figure 1 and Supplementary Figure S2).

### 2.2. Cell Proliferation

**Figure 2:**
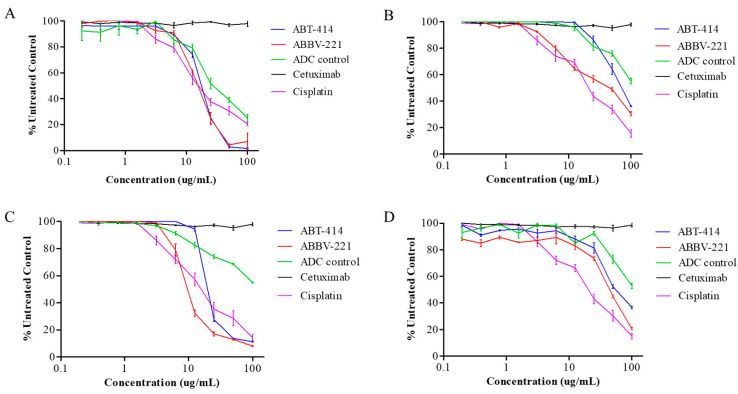
Cell proliferation assays (MTS). Cell proliferation ratio (% untreated control) and the concentration of each drug are indicated on the vertical and horizontal axes. Inhibition of cell proliferation in (**A**) MSTO-211H, (**B**) NCIH-28, (**C**) NCIH-2052 and (**D**) NCIH-2452 treated with different concentrations of ABT-414 (in blue), ABBV-221 (in red), antibody drug conjugate-microtubule inhibitor, monomethyl auristatin F (ADC-MMAF) control (in green), cetuximab (black) and cisplatin (purple) measured at day 7 of growth.

ABT-414 and ABBV-221 resulted in significant tumor growth inhibitions compared to cetuximab (*p* < 0.05) and had equivalent efficacies compared to cisplatin (Figure 2). Antitumor effects of ABT-414 and ABBV-221 were seen at drug concentrations of 5–35 µg/mL in all cell lines evaluated. Consistent with prior studies, we found that the cell lines evaluated were cetuximab-resistant [25].

### 2.3. In Vivo Therapy Studies with mAb806-Based ADCs in Mesothelioma Xenograft and PDX Tumor Models

**Figure 3:**
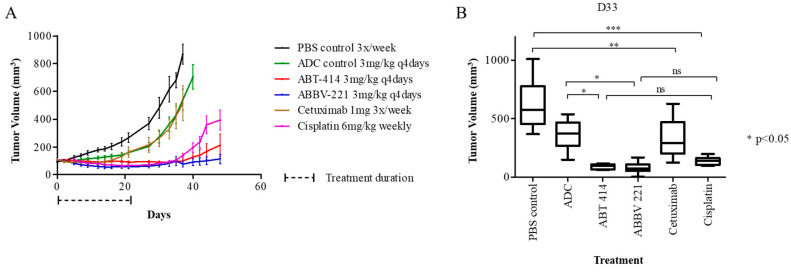
Antitumor activity of ABT-414 and ABBV-221 against the MSTO-211H xenograft model. (**A**) Tumor growth curves of mice (*n* = 10) in each group treated at the doses indicated. Significant antitumor responses to both ABT-414 (red line) and ABBV-221 (blue line) were demonstrated and superior to cisplatin (purple line). (**B**) Average tumor volumes 33 days after various treatments were initiated. There was significant tumor growth suppression in the ABT-414 and ABBV-221 treatment groups on day 33 post-therapy compared to the ADC-MMAF control group and cisplatin group. *** *p* < 0.001, ** *p* < 0.01 and * *p* < 0.05; ns: nonsignificance.

The efficacy of ABT-414 and ABBV-221 were evaluated in the MSTO-211H xenograft model based on its positive EGFR and mAb806 epitope expression. Treatment with mAb806-ADCs resulted in significant tumor growth inhibition compared to the ADC control (*p* < 0.05). The marked antitumor response was sustained in the ABT-414 and ABBV-221 treated groups 19 days following treatment cessation. At the end of treatment, day 21, ABT-414 and ABBV-221 showed similar antitumor efficacies as cisplatin (mean tumor volumes 91.3, 61.7 and 70.5 mm^3^, respectively; *p* = 0.46). However, mice treated with mAb806-ADCs had significantly longer survival until the need for euthanasia for the study endpoints compared to the cisplatin-treated mice (*p* < 0.05) and the ADC-MMAF control group (*p* < 0.0015) (Figure 3). The median survival of mice in the ABT-414 and ABBV-221 arms were 81 days and 100 days, respectively, vs. 48 days for mice treated with the ADC control and 54 days for the cisplatin-treated mice.

In addition, we assessed the EGFR and mAb806 epitope expression in PDX models and compared to the original patient sample. EGFR amplification was not seen in any tumor sample, consistent with other studies [26].

**Figure 4:**
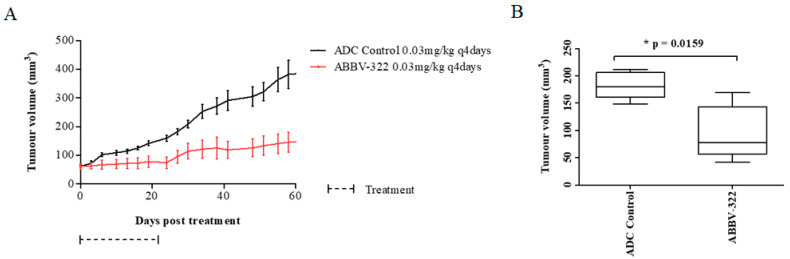
Tumor growth response and survival curves for the 14R091 epithelioid mesothelioma patient-derived xenograft (PDX) model. (**A**) Tumor growth curve of mice treated with ABBV-322 and the ADC control at the doses specified (*n* = 5 mice in each group). (**B**) Average tumor volumes from mice at 27 days after treatments were initiated. There was a statistically significant tumor growth inhibition in the ABBV-322 treatment arm on day 27 post-therapy (*p* = 0.0159, two-sided) compared to the ADC-pyrrolobenzodiazepine (PBD) control arm. The average tumor volumes were 183 mm^3^ (ADC control) and 96 mm^3^ (ABBV-322).

The efficacy of next-generation ADC ABBV-322 was then evaluated in MM PDX models. We established that the histologies of the primary tumors were concordant to those of the xenograft tumors and were maintained with multiple passages, consistent with the previous literature [27]. 14R091 is a mesothelioma PDX model of the epithelioid subtype, which has high EGFR and mAb806 epitope expression by IHC (EGFR: H score > 200 and mAb806: H score 170) but lacks true *EGFR* amplification on Fluorescence in situ hybridization (FISH). Both the PDX model and the original patient samples showed consistency in EGFR and mAb806 IHC expression (Supplementary Figure S3). ABBV-322 resulted in significant tumor growth inhibition compared to the ADC-PBD control (Figure 4). At the end of the treatment (day 27), the average tumor volumes were 183 mm^3^ ± 22 (ADC control) vs. 96 mm^3^ ± 11 (ABBV-322) (*p* = 0.016). The median survival of mice in the ABBV-322 arm was significantly longer compared to the ADC control group (150 vs. 122 days, respectively; *p* = 0.018).

**Figure 5:**
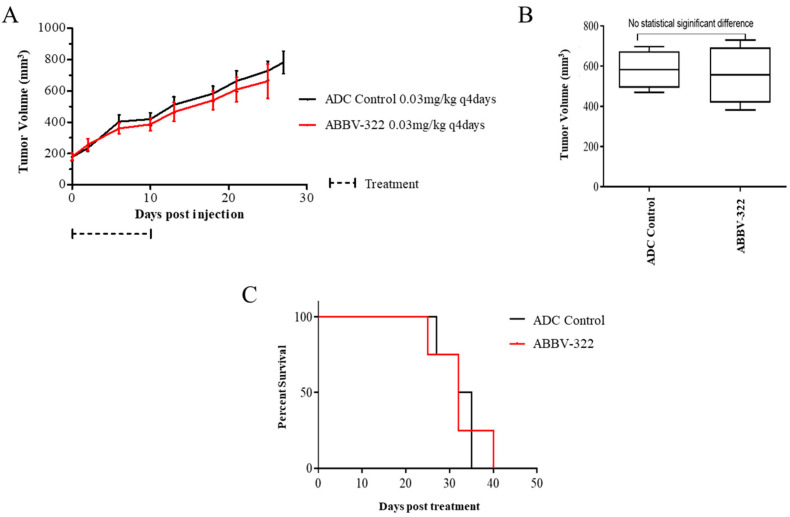
Tumor growth response and survival curves for the mAb806 epitope negative biphasic mesothelioma PDX model (MPM36). The treatments were administered at the doses indicated in the figure. (**A**) The mAb806 IHC negative PDX model demonstrated no significant tumor growth suppression of ABBV-322 compared to the ADC control group. (**B**) The differences in tumor columns between the 2 groups on day 21 post-treatment commencement were found to be nonsignificant (*p* = 0.7649, two-sided). (**C**) The survival curves showed no significant differences between the different treatment groups.

The efficacy of ABBV-322 was also evaluated in a PDX model (MPM36) of biphasic histology with high EGFR expression (H score 150) and absent mAb806 epitope staining (H score < 5). EGFR overexpression was not associated with true *EGFR* amplification. There was no significant tumor growth inhibition by ABBV-322 compared to the ADC control and no significant difference in the median survivals for both treatment groups (Figure 5).

### 2.4. Molecular Imaging and Quantitation of 806 Expression in MSTO-211H Mesothelioma Xenograft Model with ^89^Zr-ch806

Quantitative biodistribution and imaging of mAb806 uptake (^89^Zr-ch806) were performed to allow the correlation of mAb806 concentrations in MM tumors and to establish ^89^Zr-806 as a noninvasive imaging biomarker of 806 expression in MM tumors.

A Scatchard analysis indicated that the ^89^Zr-ch806 had a Ka of 0.376 × 10^9^ M^−1^, and the number of antibody binding sites per cell was 2.5 × 10^6^. In a single-point binding assay, ^89^Zr-labeled ch806 showed high and specific binding to mAb806-expressing U87MGdel2-7 cells (96.9%) and low binding to 806-negative U87MG cells (0.326%).

Stability data for ^89^Zr-ch806 after two days in human serum showed that the radiochemical purity remained high at 99.6%, and the immunoreactivity decreased to 89.1%. After seven days in human serum, the radiochemical purity decreased to 21.9%, and the immunoreactivity decreased to 14.3%.

**Figure 6:**
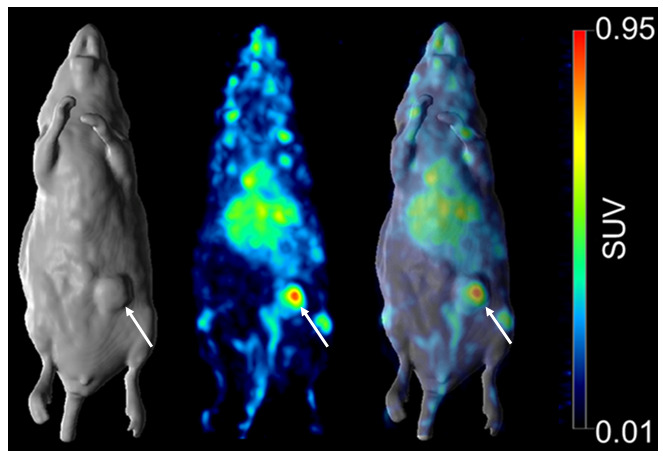
PET/MR imaging of ^89^Zr-ch806 on day 7 post-injection in a MSTO-211H xenograft-bearing BALB/c nude mouse (from left to right: whole-body MR (surface rendered), PET (maximal intensity projection) and fused PET/MR). Tumor-specific uptake (arrow), and some liver, spleen and bone uptakes, are demonstrated.

^89^Zr-ch806 demonstrated selective tumor uptake in 806-positive MSTO-221 tumors in BALB/c *nu*/*nu* mice, with normal tissues demonstrating clearance patterns typical of a radiolabeled intact humanized antibody. The tumor uptake reached 7.02 + 2.18 injected dose per gram tissue (%ID/g) on day 7. PET imaging showed a high uptake of ^89^Zr-ch806 in the tumors (Figure 6), and some bone, spleen and liver uptakes were observed, most likely due to the blood pool activity and catabolism of the free ^89^Zr-chelate.

## 3. Discussion

We demonstrated that a range of drug-conjugated mAb806 ADCs targeting a tumor-specific epitope of EGFR had significant efficacy in MM models. Treatments with ABT-414, ABBV-221 and ABBV-322 resulted in significant tumor growth inhibition in EGFR-overexpressing cell lines in vitro, as well as in their derived xenografts and PDX models with high EGFR and mAb806 epitope expression. In contrast, in an EGFR-expressing PDX model where exposure to the mAb806 epitope was absent, no growth inhibition was observed.

Previous preclinical studies have evaluated the use of radiolabeled anti-EGFR antibodies using a variety of radioconjugates, with mixed results ([4,28,29]. This study demonstrated the specific targeting of mAb806-expressing MM tumors using ^89^Zr-based PET imaging. ^89^Zr-ch806 was stable in the serum at 37 °C with a retention of immunoreactivity, radiochemical purity and construct integrity for up to seven days and showed targeting of 806-positive tumors on PET/MRI imaging. The splenic and bone uptakes seen with ^89^Zr-ch806 were a result of catabolized free ^89^Zr and similar to that seen with other ^89^Zr-labeled antibodies [30,31,32]. Similarly, levels of tracer uptake in the liver were consistent with other studies evaluating other radioconjugates conjugated to anti-EGFR antibodies [24,29].

The ability of ADCs to deliver highly potent payloads in the targeted cancer cell (e.g., with EGFR expression) with reduced off-target toxicity makes this approach very attractive in the management of MM. We have previously reported that mAb806-ADC has markedly superior antitumor efficacy in animal models compared to antibody alone and the drug alone at similar concentrations [21]. The ability of mAb806 ADCs to target a conformational epitope exposed in tumor-specific conditions allows effective tumor targeting without the conventional EGFR inhibitor-mediated toxicities [13]. MAb806 ADCs were shown to retain affinity to the mAb806 epitope comparable to a nonconjugated antibody, thus ensuring a high tumor uptake [15,18]. This approach was successful in the management of glioblastoma and demonstrated efficacy in a range of treatment refractory-advanced solid tumors with an acceptable toxicity profile [19,20,33]. Our data suggest that targeting EGFR in MM using specific ADCs is a valid therapeutic strategy and supports the further investigation of mAb806 epitope expression as a predictive biomarker to select for patients that are most likely to respond to this novel class of ADCs in MM. One such approach could be the use of ^89^Zr-ch806 PET imaging allowing the real-time evaluation of mAb806 epitope expression and the detection of inter-lesional heterogeneity prerequisites for clinical benefit from ADC treatment. Our results also confirm that the deliverable concentration of mAb806 to MM can be measured by PET imaging and may assist with comparing the ADC concentration in tumors to therapeutic responses in clinical trials [34].

Although the therapeutic approach of using ABT-806-based ADCs in MM with EGFR and mAb806 expression appears to be novel and appealing, one must remain cautious, as there have been other protein-specific targeted therapies previously trialed in MM that did not show subsequent clinical efficacy, despite promising preclinical data. Mesothelin-targeted therapies involving anti-mesothelin immunotoxins (SSP1), chimeric anti-mesothelin antibodies (amatuximab) and mesothelin-directed antibody-drug conjugates (anetumab ravtansine) have shown phase I efficacy, but these were not replicated in the later phase studies [35,36,37]. It remains uncertain why the anti-mesothelin antibody approach did not work in MM despite mesothelin being present in a majority of MM patients and whether a different cytotoxic payload may have led to a difference in the sensitivity and efficacy. The question also remains with regards to the possible efficacy of these agents in combination with chemotherapy or immunotherapy. However, the interest dwindled, given that monotherapy did not show significant efficacy. Observations from the anti-mesothelin antibody studies could provide new insight and perspectives for the future development of targeted agents in MM. The identification of robust predictive biomarkers would be crucial to determine which subset of patients respond to these targeted therapies in MM. In this context, the ability to image the 806 conformational epitopes in EGFR-positive tumors, as demonstrated in our data, may represent one approach to identifying MM patients suitable for treatment, although this would require more careful exploration in clinical trials.

However, there are a few limitations to our study that should be acknowledged. PDX models are favored to cell line xenograft models, as they more accurately recapitulate the molecular characteristics or behaviors present in patient tumors [38]. However, they are also more difficult to develop and often generate tumors of heterogenous sizes, making it more difficult to control these models in a therapeutic experiment compared to cell line xenograft models. Orthotopic models are more similar to the original tumors in patients, as they recapitulate the tumor microenvironments of the transplanted tumors in their originating sites [39,40,41]. However, orthotopic PDX models are technically challenging in MM, and not all cell lines will easily grow in the pleural cavity. The MM models used for this study do provide evidence for the efficacy of 806-based ADCs in vivo, despite these caveats.

## 4. Materials and Methods

### 4.1. Antibodies, ADCs and Chemotherapeutics

ABT-806 was produced by the transient transfection of HEK-293-6E cells, as described previously [42]. Maleimidocaproyl (mc) monomethyl-auristatin F (MMAF) and monomethyl-auristatin E (MMAE) to generate ABT-414 and ABBV-221, respectively, were provided by Seattle Genetics. A humanized immunoglobulin-1 (HuIgG1)—conjugated to the cytotoxic payload MMAF or PBD—was used as a negative ADC control. IgG control antibody was produced in-house. Cisplatin and cetuximab (Bristol-Myers Squibb) were purchased. Stock solutions were prepared according to each drug’s specific instructions and stored at −20 °C. Drugs were diluted in fresh media before each experiment.

### 4.2. Cell Cultures

The MM cell lines NCI-H28 and NCI-H2052 (sarcomatoid), NCI-H2452 (epithelioid) and MSTO-211H (biphasic) were purchased from the American Type Culture Collection and maintained in RPMI-1640 medium supplemented with 10% fetal calf serum (FCS) (Invitrogen, Thermo Fisher Scientific Inc., Waltham, MA, USA). The tumor cell lines U87MG and U87MG.Δ2-7, a derivative of U87MG transfected with EGFRvIII [43], were maintained in DMEM medium supplemented with 10% FCS. In addition, the transfected cell line was maintained in 400 µg/mL of Geneticin. All cell lines were authenticated by short tandem repeat (STR) authentication and confirmed as mycoplasma-negative.

### 4.3. FACS Analysis

EGFR expression levels in cell lines were confirmed by flow cytometry. Cells (4 × 10^4^) were incubated for 1 hour with 10-μg/mL mAb806, mAb528 or IgG1 isotype control antibody. Mab528 is a pan-EGFR antibody with a similar binding profile to cetuximab [44]. Cells were then incubated for 1 h with Alexa 488-conjugated anti-mouse IgG antibody and analyzed on a BD FACS Canto II flow cytometer (BD Biosciences, Piscataway, NJ, USA).

### 4.4. Western Blot

In brief, cell lysates were subjected to sodium dodecyl sulfate polyacrylamide gel electrophoresis and immunoblotted with antibodies against EGFR and mAb806 epitope. The primary antibodies used included rabbit anti-human EGFR (#4267, Cell Signalling, Danvers, MA, USA) at 1:1000 dilution, mouse anti-human EGFR-806:mAb806 (Ludwig Institute for Cancer Research (LICR), Olivia-Newton John Cancer Research Institute (ONJCRI)) at 3 mg/mL and mouse anti-human actin (#3700, Cell Signalling, Danvers, MA, USA) at 1:1000 dilution. The secondary antibodies included goat anti-rabbit Ig (A2554, Sigma-Aldrich, St. Louis, MO, USA) at 1:3000 dilution and goat anti-mouse Ig (A6154, Sigma-Aldrich, St. Louis, MO, USA) at 1:3000 dilution.

### 4.5. Cytotoxicity Assays

Cells lines were plated at 1–3 × 10^3^ cells per well in complete growth medium containing 10% FCS in 96-well plates and allowed to adhere overnight. Cells were treated with cetuximab, cisplatin or ADCs in a 1:3 dilution to a maximum dose of 100 μg/mL. Proliferation was assessed with the MTS assay (Sigma-Aldrich, St. Louis, MO, USA), as previously described [45].

### 4.6. Immunohistochemistry Analysis

EGFR expression was evaluated by immunohistochemical (IHC) staining. Slides were deparaffinized and rehydrated. Antigen retrieval was achieved, and endogenous peroxidase and nonspecific protein-binding sites were blocked using 3% hydrogen peroxide solution and subsequently incubated in the primary antibody: EGFR (31G7 clone; Zymed/Invitrogen) at 2.5 µg/mL and mAb806 (LICR BDF; in-house) at 5 µg/mL. Novolink Kit Poly-HRP secondary antibody was used with diaminobenzidine (DAB) as the chromogen (Dako, Santa Clara, CA, USA) and counterstained with hematoxylin (Dako, Santa Clara, CA, USA). Samples staining ≥ 5% of the malignant cells were considered positive and scored 0 to 3+ by one investigator and pathologist for validation. An “H score” was calculated based on the intensity and percentage of cells stained [46].

### 4.7. In Vivo Studies

Female NOD scid gamma (NSG) and BALB/c *nu*/*nu* mice were obtained from the Bio-Resource Facility at Austin Health, Melbourne, VIC, Australia. All animal studies were approved by the Austin Hospital Animal Ethics Committee, Melbourne, VIC, Australia and were conducted in compliance with the Australian Code for the care and use of animals for scientific purposes and were performed as previously described [18].

To establish xenografts, 2 × 10^6^ MSTO-211H cells mixed with 75-uL Matrigel (BD Sciences, Piscataway, NJ, USA) were injected subcutaneously in the right flank of 5 to 6-week-old female BALB/c *nu*/*nu* mice. MM PDX models previously established at Princess Margaret Hospital (PMH Cancer Centre), Toronto, ON, Canada) [27] were used for in vivo experiments. Prior to use in this study, tumor histology and STR authentication with parent tumors were performed for all PDX models. To generate patient-derived xenografts, ~5-mm patient tumor samples mixed with 20 µL of Matrigel (BD Biosciences, Piscataway, NJ, USA) were implanted into the flanks of 6-8-weeks-old NSG mice. Once the tumors were engrafted, the tumors were further passaged after the histology was confirmed by a pathologist. All PDXs were evaluated for EGFR and mAb806 epitope expression by IHC and Western blot.

Intraperitoneal treatments were commenced once tumors reached a mean volume of ~100 mm^3^ and administered three times per week for a total duration of three weeks. Tumor measurements were taken two to three times per week by electronic calipers and calculated using the formula V = L × W^2^/2, where “W” represents the width of the tumor and “L” the length of the tumors. For the MSTO-211H study, mice received either ABT-414, ABBV-221 or ADC control (3 mg/kg) every 4 days; cetuximab (1 mg) 3 times per week; cisplatin chemotherapy (6 mg/kg) once per week or PBS (100 µL) 3 times per week. For the PDX 14R091 and PDX MPM36 studies, mice received ABBV-322 (0.03 mg/kg) or control ADC (0.03 mg/kg) every 4 days, for a total of 12 treatments.

Mice were euthanized when the tumor volume reached a maximum of 1000 mm^3^, significant weight loss (>10% of body weight within 3 days) or other morbidities or reached the clinical end-point (overall survival), whichever occurred first, as per our institutional ethics guidelines.

### 4.8. Chelation and Radiolabeling of ^89^Zr-Labeled-ch806

To determine the quantitative targeting of MM by mAb806 in tumor models, a ^89^Zr-labeled immunoconjugate was developed. The chelation, radiolabeling and in vitro characterization of ^89^Zr-labeled antibody ch806 was performed as previously described by our group [34]. Analytic-grade reagents, a sterile technique and pyrogen-free plasticware were used in all labeling steps. Chimeric anti-EGFR antibody ch806 was chelated with the bifunctional metal ion chelator *p*-isothiocyanatobenzyldesferrioxamine (Df; Macrocyclics Inc., Dallas, TX, USA) at a 3.0-fold molar excess, as previously described [47]. Following chelation, Df-ch806 was trace-radiolabeled as follows: a solution containing 99.9 ± 16.1 MBq (2.7 mCi) of positron-emitting ^89^Zr (PerkinElmer, Melbourne, VIC, Australia) was mixed with 1.0-mg Df-ch806 for 45 minutes. The mixture was then quenched with EDTA. The product was purified on a Sephadex G50 column (Pharmacia, Uppsala, Sweden) equilibrated with sodium chloride BP 0.9% *w*/*v* (Pfizer, Sydney, Australia), with resultant radioconjugates assessed for radiochemical purity and immunoreactivity.

### 4.9. Quality Control and In Vitro Characterization of ^89^Zr-Labeled-ch806

The amount of free vs. bound antibody after radiolabeling was determined by instant thin-layer chromatography using silica gel impregnated glass fiber strips (Gelman Sciences, Ann Arbor, MI, USA). Assays were performed in duplicate. Radioactivity was measured with an automated γ-counter (Wizard; Perkin Elmer, MA, USA). The immunoreactive fraction of the radiolabeled ch806 constructs with 806-positive U87MG.Δ2-7 cells was determined by linear extrapolation to binding at an infinite antigen excess using a Lindmo assay [48], as previously described [49]. Scatchard analysis was used to calculate the apparent association constant and number of antibody molecules bound per cell [49]. Serum stability was assessed by incubating 20-μg ^89^Zr-Df-ch806 in 100 μL of human serum at 37 °C for a 7-day period. Radiochemical purity and single-point immunoreactivity assays at 0 (day of radiolabeling, no incubation), 3 and 7 days of incubation were performed with U87MG.Δ2-7 cells and the U87MG control cell line (5 × 10^6^) under conditions of antigen excess.

### 4.10. Biodistribution and Imaging Study with ^89^Zr-Labeled-ch806

BALB/c *nu*/*nu* mice bearing established MSTO-211H tumors were injected intravenously with 1.99MBq (53 µCi) of ^89^Zr-ch806. On day 0 (2 h), 2 and 7, post-injection mice were sacrificed by the over-inhalation of isoflurane anesthesia, and the biodistribution of radiolabeled ch806 was assessed. PET imaging was performed on day 2 and day 7 post-injection in two animals using a small animal nanoPET/MR hybrid imaging system (nanoScan® PET/MR camera, Mediso, Budapest, Hungary). All PET raw data were dead time, random and attenuation-corrected. The volumetric images were reconstructed with a transaxial matrix size of 255 × 255, using the built-in quasi Monte Carlo simulation algorithm combined with filtered sampling and stochastic iteration.

### 4.11. Statistical Analysis

The IC_50_ and EC_50_ values were determined by a nonlinear regression analysis of the concentration response curves using GraphPad Prism 6.0. Data from experiments in vivo were analyzed using the one-way ANOVA with post hoc Bonferroni correction for TGI_max_ and the Mantel–Cox log-rank test for TGD (GraphPad Prism, GraphPad Software, San Diego, CA, USA).

## 5. Conclusions

In conclusion, our data showed promise in utilizing these agents either alone or in combination, especially given that their safety has already been demonstrated in other tumor types. For a disease where no changes in therapeutic paradigms have been appreciated in over 20 years, the promise of novel strategies is both important and timely.

## Supplementary Materials

The following are available online at https://www.mdpi.com/1424-8247/13/10/289/s1: Figure S1: ABBV-322 structure and function. Figure S2: EGFR and mAb806 epitope expression by IHC of PDX and original patient tumor sample. Figure S3: EGFR (top row) and mAb806 epitope (bottom row) IHC staining of the 14R091 primary patient MM tissue sample (left) and the corresponding PDX tumor (right) demonstrates consistence in EGFR and 806 IHC expressions.
