# Supplementary material for: Targeting and Efficacy of Novel mAb806-Antibody-Drug Conjugates in Malignant Mesothelioma
Source: Pharmaceuticals (Basel). 2020 Oct 2;13(10):289. doi: 10.3390/ph13100289 (PMC7601847; doi:10.3390/ph13100289)
Supplement: Supplementary file 1 [file pharmaceuticals-13-00289-s001.pdf]

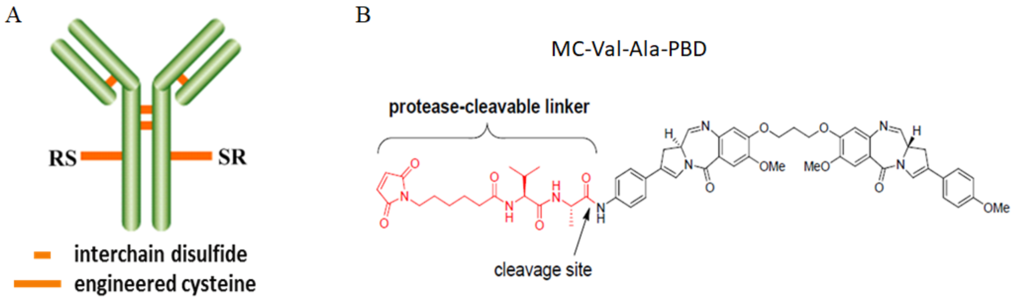

Supplementary Figure S1. ABBV-322 consists of A) ABT-806 S238C; B) a cathepsin-cleavable Val-Ala linker and a PBD dimer (SGD-1882). Two Mc-Val-Ala-PBD drug-linker molecules are conjugated to the cysteine-engineered ABT-806 S238C at position 238 for a DAR (drug antibody ratio) of two.

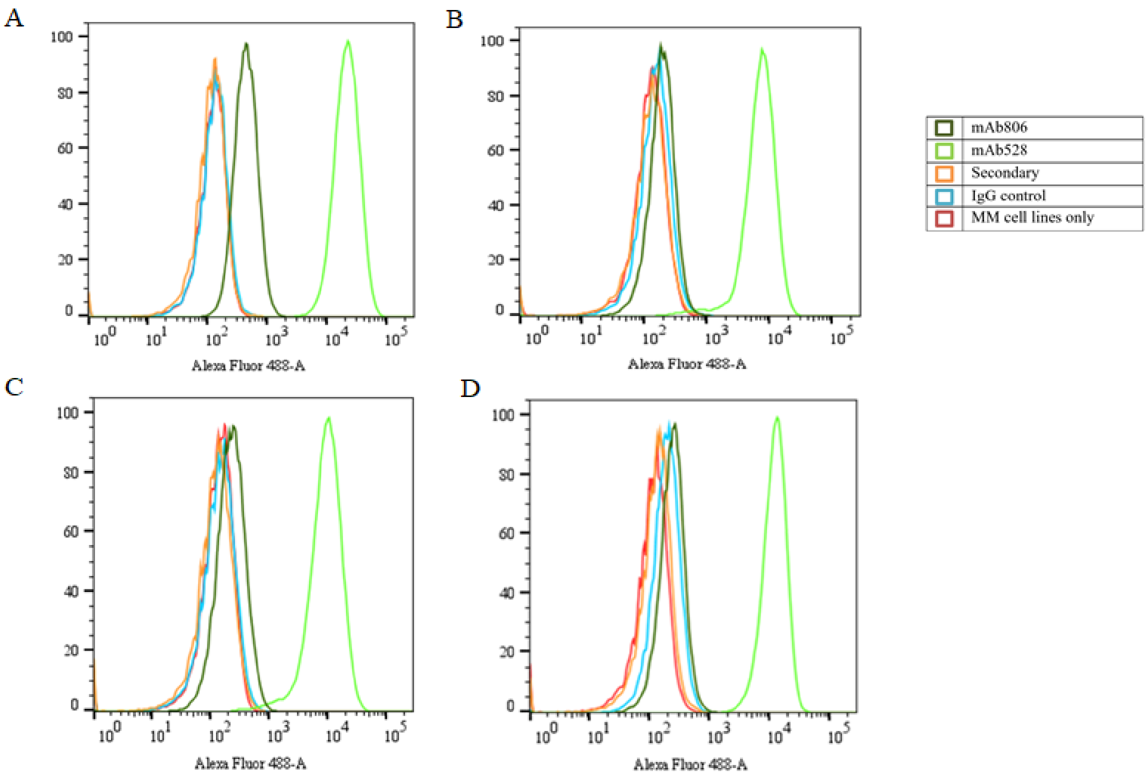

Supplementary Figure S2. EGFR and mAb806 expression in MM cell lines using flow cytometry analysis. Histogram overlay showing the fluorescence shift between wtEGFR (mAb528), mAb806, secondary antibody, muIgG idiotype control, and cells only control in A) NCIH-28, B) NCIH-2052, C) MSTO 211H and D) NCIH 2452 cells .

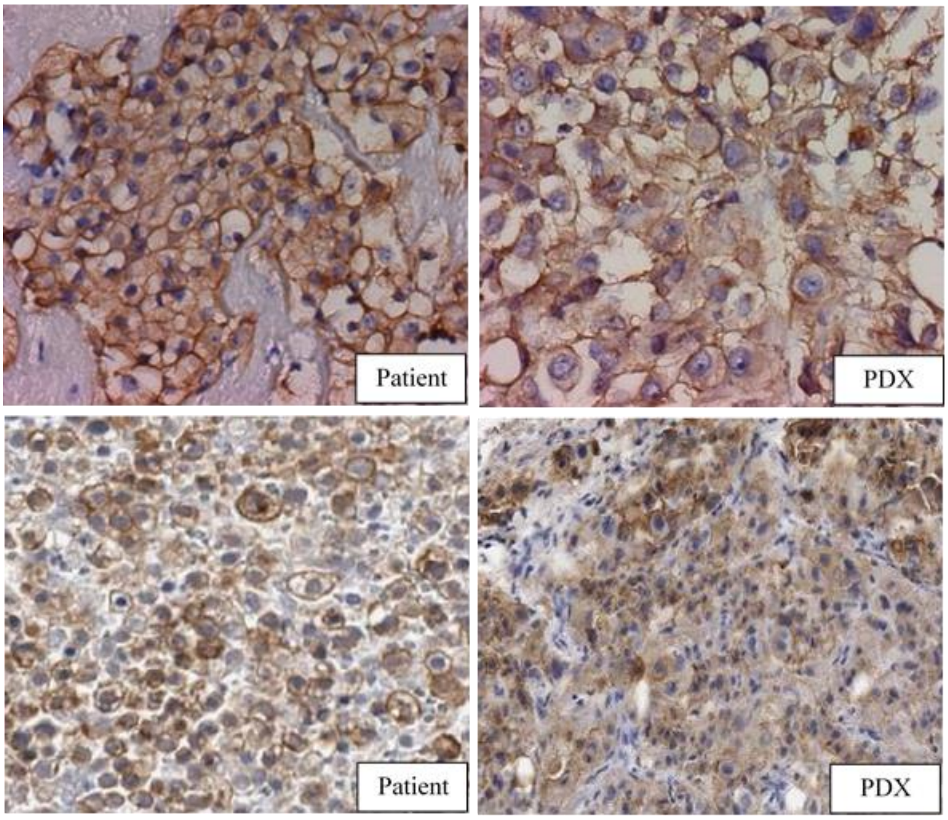

Supplementary Figure S3: EGFR (top row) and mAb806 epitope (bottom row) IHC staining of the 14R091 primary patient MM tissue sample (left) and the corresponding PDX tumor (right) demonstrates consistence in EGFR and 806 IHC expressions.
